# The Immune Response to Eastern Equine Encephalitis Virus Acquired Through Organ Transplantation

**DOI:** 10.3389/fmicb.2020.561530

**Published:** 2020-09-24

**Authors:** Vanessa Raabe, Lilin Lai, Yong Xu, Chris Huerta, Dongli Wang, Stephanie M. Pouch, Crystal W. Burke, Ashley E. Piper, Christina L. Gardner, Pamela J. Glass, Mark J. Mulligan

**Affiliations:** ^1^Hope Clinic of the Emory Vaccine Center, Division of Infectious Diseases, Department of Medicine, School of Medicine, Emory University, Atlanta, GA, United States; ^2^Division of Infectious Diseases, Department of Medicine, School of Medicine, Emory University, Atlanta, GA, United States; ^3^Virology Division, United States Army Medical Research Institute of Infectious Diseases, Frederick, MD, United States

**Keywords:** eastern equine encephalitis virus, adaptive immunity, cellular immunity, humoral immunity, eastern equine encephalomyelitis

## Abstract

The human immune response to eastern equine encephalitis virus (EEEV) infection is poorly characterized due to the rarity of infection. We examined the humoral and cellular immune response to EEEV acquired from an infected donor via liver transplantation. Both binding and highly neutralizing antibodies to EEEV as well as a robust EEEV-specific IgG memory B cell response were generated. Despite triple-drug immunosuppressive therapy, a virus-specific CD4+ T cell response, predominated by interferon-γ production, was generated. T cell epitopes on the E2 envelope protein were identified by interferon-γ ELISpot. Although these results are from a single person who acquired EEEV by a non-traditional mechanism, to our knowledge this work represents the first analysis of the human cellular immune response to EEEV.

## Introduction

Eastern equine encephalitis virus (EEEV), an alphavirus in the *Togaviridae* family, is a rare arboviral infection in North America with only 121 human cases reported in the United States from 2003 to 2016 (Lindsey et al., 2018). However, EEEV is an emerging threat in the United States as thirty-six human cases of EEE, including fourteen fatalities, were reported in 2019, representing a significant increase in symptomatic human infection compared to previous years (Morens et al., 2019). Most humans infected with EEEV are asymptomatic or develop a non-specific febrile illness while a minority develop encephalitis, which is associated with high rates of hospitalization and mortality in 41% of cases (Goldfield et al., 1968; Lindsey et al., 2018). Due to the rarity of infection, the cellular immune response to EEEV infection has never been described in humans and only limited humoral response data exists.

Although usually transmitted by mosquitoes, a series of three cases of EEEV infection among organ transplant recipients secondary to transmission from a single infected donor in 2017 was recently described; EEEV-related complications contributed to the death of two of the transplant recipients (Pouch et al., 2018). We describe the humoral and cellular immune response to EEEV in the liver transplant recipient, who was a 40 year old woman with a history of autoimmune hepatitis who received her second liver transplant from the donor retrospectively identified as infected with EEEV. The organ donor had detectable EEEV RNA in the blood at the time of organ collection but no detectable EEEV antibodies (Pouch et al., 2018). The liver recipient subsequently developed fevers the day following transplant and confusion 6 days following transplant leading to obtundation due to encephalitis with multiple non-enhancing regions of restricted diffusion in the basal ganglia, temporal lobes, and thalami on MRI (Pouch et al., 2018). EEEV was diagnosed on day 8 after transplantation based on positive EEEV IgM antibodies in cerebrospinal fluid. She had poor neurological recovery with repeat imaging demonstrating cerebral vasculitis and died 3 months post-transplantation (Pouch et al., 2018).

## Materials and Methods

### Study Approval

Written consent for study participation was obtained from the family of the study patient using an Emory University Institutional Review Board approved protocol for phlebotomy for infectious diseases of public health importance.

### Safety, Collection, and Processing

For biosafety reasons, we utilized only blood samples collected prior to infection or after the clearance of viremia was documented by molecular testing on day of infection (DOI) 27. Whole blood, serum, plasma, and peripheral blood mononuclear cells (PBMCs) were collected. Blood for PBMC separation was collected on DOI 48 using CPT^TM^ tubes with sodium heparin (BD #362753) and processed within 2 h of collection under biosafety level (BSL) 2+ conditions. Processing included harvesting of PBMCs after CPT^TM^ tube centrifugation, washing with PBS, and cryopreservation in 90% fetal bovine serum (FBS) with 10% DMSO using a StrataCooler (Agilent) at −80°C. PBMCs were stored in liquid nitrogen prior to use. Residual frozen sera and plasma from clinical testing were obtained from the day prior to transplant (DOI −1) and from DOI 39.

### ELISA

Levels of EEEV-specific binding IgG and IgM antibodies were assessed by indirect ELISA using β-propiolactone-inactivated eastern equine encephalitis suckling mouse brain antigen (EEEV antigen) provided courtesy of the Arbovirus Reference Collection of the Centers for Disease Control and Prevention (CDC). In brief, Nunc MaxiSorp plates (Fisher #439454) were coated with antigen diluted 1 in 500 with phosphate-buffered saline (PBS) and incubated overnight at 4°C. Plates were blocked for 1 h using PBS containing 0.05% Tween-20 (PBS-T), 5% dry milk, and 4% whey. Diluted serum was added in three-fold dilutions starting at 1:10, incubated for 1 h, washed with PBS-T, and incubated with horseradish peroxidase goat anti-human IgG antibody (Jackson ImmunoResearch #109-036-098) at 1:20000 for 1 h. KPL SureBlue^TM^ TMB Microwell Peroxidase Substrate (KPL 52-00-00) was added for 5 min, the reaction was stopped with 1N hydrochloric acid (VWR #BDH3202-2), and optical densities at 450 nm were read using a BioTek EL808 ELISA plate reader at room temperature. Positive thresholds for IgG and IgM end point titer calculations were derived from the geometric mean optical density plus twice the standard deviation of optical densities obtained on DOI −1 to EEEV antigen and to normal suckling mouse brain antigen, provided courtesy of the Arbovirus Reference Collection of the CDC, at all time points. Samples were run in duplicate at all time points under BSL 2+ conditions.

### Neutralizing Antibodies

*In vitro* neutralization was measured using a plaque reduction neutralization test (PRNT) under BSL 3 conditions. Briefly, serum samples were heat-inactivated for 30 min at 56°C. Serum samples were diluted 1:10 in Minimum Essential Medium with 2% heat-inactivated FBS, 1% HEPES, and 2% Penicillin/Streptomycin and then serially diluted 1:2. EEEV FL93-939, VEEV Trinidad donkey, or WEEV CBA87 virus stocks were diluted to a concentration of 2.0 × 10^3^ PFU/ml and added 1:1 to the serially diluted samples or control well containing media alone for the virus only control. All samples were incubated overnight at 2–8°C. Six-well plates of Vero 76 cells were grown to ∼90–100% confluence. Cells were infected with 0.1 mL of each serial dilution per well in duplicate. Plates were incubated at 37 ± 2°C for 1 h ± 15 min with gentle rocking every 15 min. After 1 h, cells were overlaid with 0.6% agarose in Basal Medium Eagle (BME) with 5% heat-inactivated fetal bovine serum (HI-FBS), and 1% Penicillin/Streptomycin, and incubated for 24 ± 4 h at 37 ± 2°C, 5 ± 1% CO_2_. A second overlay containing 0.6% agarose in BME with 5% HI-FBS, 1% Penicillin/Streptomycin, and 5% of total volume neutral red vital stain was added to wells and further incubated ∼18–24 h for visualization of plaques. Plaques were counted following incubation with stain overlay. The virus only control was counted and the endpoint titer was determined to be the highest dilution with ≥80% reduction (PRNT_80_) in the number of plaques observed relative to virus-only control wells.

### Immune Cell Phenotyping

Immune cell phenotyping was performed under BSL 2+ conditions by incubating 200 μL of fresh whole blood in polystyrene tubes with two different fluorochrome-labeled antibody panels for 20 min in the dark [Panel 1: anti-CD3 (SP34-2, #562877), anti-CD4 (L200, #560836), anti-CD8 (SK1, #341051); anti-CD19 (HIB19, #555415), anti-CD38 (HIT2, #555460), and anti-HLA-DR (G46-6, #555811) from BD, anti-CD20 (2H7, #47-0209-42) from eBioscience, and anti-CD27 (O323, #302838) from BioLegend. Panel 2: anti-CD3 (UCHT1, #557943), anti-CD11c (O33-782, 561355), anti-CD14 (M5E2, #565283), anti-CD19 (HIB19, #557921), anti-CD123 (7G3, #554529), and anti-HLA-DR (G46-6, #560651) from BD; anti-CD16 (CB16, #47-1068) and anti-CD56 (MEM188, #17-0569) from eBioscience, and anti-CD20 (2H7, #302332) from BioLegend]. Flow cytometry was performed on an LSRII (BD) and data was analyzed using FlowJo software version 9 (Tree Star). T cells expressing both HLA-DR and CD38 were considered activated.

### PBMC Thawing

Cryopreserved PBMCs were thawed in warmed sterile, complete RPMI (R10) media containing DNase I (Roche #04716728001), washed, and resuspended in R10 with DNase I under BSL 2+ conditions. Cell counting and viability assessment was performed using a Guava easyCyte counter (Luminex) per the manufacturer protocol. Freshly thawed cells were rested overnight in an incubator at 37°C with 5% carbon dioxide and cells were recounted the following day yielding >85% viable cell recovery.

### Memory B Cell ELISpot

ELISpot assays for memory B cells producing IgG to EEEV antigen, normal suckling mouse brain antigen, or recombinant chikungunya virus glycoprotein (CTK Biotech, #A2321) were performed as previously described under BSL 2+ conditions (Lai et al., 2018). Memory B cell ELISpots were performed in triplicate for each antigen.

### Intracellular Cytokine Staining

Intracellular cytokine staining was performed under BSL 2+ conditions on PBMCs collected on DOI 48 to assess for production of interferon γ (IFN-γ), interleukin 2 (IL-2), macrophage inflammatory protein 1β (MIP-1β), and tumor necrosis factor α (TNF-α) in response to stimulation with EEEV antigen, normal suckling mouse brain antigen, or phorbol 12-myristate 13-acetate (PMA) plus ionomycin (eBioscience # 00-4970-03; positive control). Stimulated cells without antigen were used as a negative control. In brief, cryopreserved PBMCs were thawed and rested overnight followed by an 18-h incubation with the target antigens, CD28 (BD #555725), and CD49d (BD #555501) at 37°C; brefeldin A and monensin (eBioscience #5537) were added after 6 h of incubation. Cells were washed, stained with Zombie Aqua viability dye (BioLegend, #L423101), and fixated/permeabilized using Cytofix/Cytoperm (BD, #554722). Staining was performed using fluorescent-conjugated antibodies to CD3 (SP34-2, #562877), CD4 (L200, #560836), CD8 (RPA-T8, #555367), IL-2 (MQ1-17H12, #554567), MIP-1β (D21-1351, #560680), and TNF-α (Mab11, #560679) from BD and IFN-γ (4S.B3, #47731942) from eBioscience. Flow cytometry was performed on an LSRII and data analysis was performed using FlowJo. A positive T cell response was defined as a level of cytokine production to EEEV antigen at least twice that of a negative control and normal suckling mouse brain antigen.

### T Cell Epitope Prediction

Predicted T-cell epitopes to EEEV envelope protein 1 (E1) and envelope protein 2 (E2) were calculated for the patient’s alleles identified by HLA-typing performed prior to organ transplantation using Immune Epitope Database and Analysis Resource TepiTool (Paul et al., 2016). Sequences for the E1 and E2 proteins from Florida strain 91–469 were obtained from UniProt accession number Q4QXJ7 (Consortium, 2018). The patient’s class I alleles were HLA-A 02/03, HLA-B 57/58, and HLA-C 03:10/06; the class II alleles were HLA-DQA1 02:01/05:01, HLA-DQB1 02:01/03:09, and HLA-DRB1 03:17/07:01. Peptide predictions (9-mer) were used for MHC-I predicted epitopes and 15-mer peptides were used for MHC-II predictions. If incomplete results were available to the specific HLA protein level, supertype prediction was used if available. If neither of these were available, the lowest available specific HLA protein value was used.

### T Cell Epitope Mapping

Cryopreserved PBMCs were thawed and rested overnight as outlined above. Fifteen-mer peptides, with 10-amino acid overlap, spanning the entire EEEV E1 and E2 proteins (provided courtesy of United States Army Medical Research Institute for Infectious Diseases) were combined into forty-eight pools ranging from 3 to 6 peptides in size at 20 μg/mL for initial epitope screening. Each peptide was present in two, non-overlapping pools. ELISpot plates (Millipore #MSIPS4510) were pre-wet for less than 1 min with 35% ethanol, washed with de-ionized water and PBS, and coated with 10 μg/mL of anti-human IFN-γ antibody (Mabtech #3420-3-250). Plates were washed with PBS, blocked with R10 media, and peptide pools in stimulation media [R10 containing 1 μg/mL each of CD28 (BD #555725) and CD49d (BD #555501)] and 2 × 10^5^ PBMCs/well on R10 were added and incubated overnight at 37°C. *Staphylococcus* enterotoxin B (Sigma #S4881) and plain stimulation media were used as controls. Plates were washed with PBS and PBS-T, incubated for 2 h with biotinylated anti-human IFN-γ (Mabtech #3420-6-250), washed with PBS-T, and incubated with Streptavidin-HRP (BD #BDB557630) for 1 h. Plates were washed and developed using 1x AEC substrate (BD # BDB551951) for 5 min, then rinsed with cold water. Spots on air dried plates were read and counted using a CTL ImmunoSpot^®^ S6 Universal Analyzer with ImmunoSpot 5.0 software. All samples were run in duplicate under BSL 2+ conditions. Peptide pools producing at least three times as many spots as the negative control wells were considered positive; individual peptides were selected for a subsequent ELISpot to further delineate T cell target epitopes if both pools containing the peptide met criteria for positivity listed above. Single peptide ELISpots were run in duplicate on the EEEV patient and a healthy control. A positive individual peptide T cell response from the EEEV patient was defined by an average number of spots at least three times the average number of spots compared to the corresponding healthy control result and the negative control.

## Results

### Serology

The day of transplantation was designated as day of infection (DOI) 1. Serum or plasma was available for EEEV serological testing from the day prior to transplant (DOI −1), DOI 39, and DOI 48. Subsequent samples could not be obtained due to the patient’s clinical condition. Prior to transplantation on DOI −1, the IgM endpoint titer was <1:20 and neither IgG antibodies nor neutralizing antibodies were detectable. At DOI 39 and DOI 48, IgM antibody endpoint titers were 1:954 and 1:812, IgG endpoint titers were 1:1405 and 1:2395, and neutralizing antibody endpoint titers were 1:20480 (Table 1). No cross-neutralizing antibodies were detectable against Venezuelan equine encephalitis virus (VEEV) or western equine encephalitis virus (WEEV; data not shown) at any time point.

**TABLE 1:** Reciprocal IgG, IgM, and neutralizing antibodies titers to EEEV.

	DOI −1 (Pre-transplant)	DOI 39	DOI 48
IgG Titer	Negative	1:1405	1:2395
IgM Titer	<1:20	1:954	1:812
Neutralizing antibodies (PRNT_80_)	<1:20	1:20480	1:20480

*Serology demonstrates no pre-existing antibodies to EEEV prior to transplant. IgG titers increased from DOI 39 to DOI 48 while IgM titers declined and neutralizing antibody titers remained stable.*

### Immune Cell Phenotyping

**FIGURE 1:**
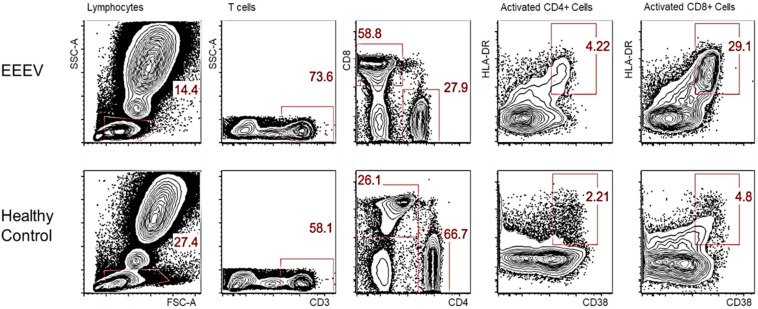
Activated CD4+ and CD8+ T Cells on DOI 48. This phenotypic assay reveals that the CD8+ T cells in the EEEV infected patient were highly activated; the CD4+ T cells were mildly activated.

Immune cell phenotyping was performed on whole blood to assess the composition of the cellular immune milieu in the setting of acute EEEV infection. Whole blood was only available on DOI 48. At DOI 48, the absolute number of total CD8+ T cells was within the normal range, however, decreased absolute numbers of total lymphocytes, CD4+ T cells, B cells, NK cells, monocytes, and dendritic cells were observed (Table 2; Autissier et al., 2010). The CD4+/CD8+ T cell ratio was inverted at 1:2.09. CD4+ T cells were mildly activated (HLA-DR+CD38+, 4.22%) and CD8+ T cells were highly activated (29.1%) (Figure 1).

**TABLE 2:** Absolute numbers of immune cells at DOI 48.

	EEEV Patient	Normal range (8) (cells/μL)
Lymphocytes	1100	1683–3068
T Cells	796	1140–1856
CD4+ T cells	224	520–1173
CD8+ T cells	468	410–825
B Cells	113	140–435
NK Cells	73	115–824
Monocytes	79	309–605
Dendritic Cells	4	35–83

*Low absolute numbers of all cell immune lines except for CD8+ T cells were present in the EEEV patient at DOI 48.*

### Memory B Cell ELISpot

**FIGURE 2:**
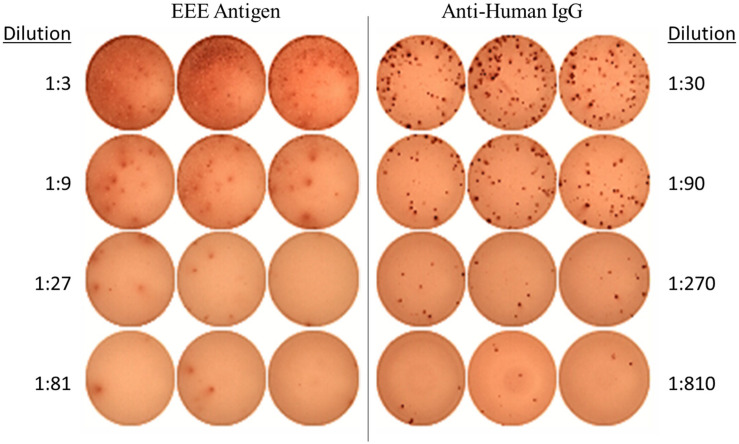
EEEV Antigen-specific Memory B Cell Response demonstrated on ELISpot Assay (DOI 48). The memory B cell ELISpot demonstrated the EEEV patient generated IgG-producing memory B cells capable of recognizing EEEV antigen.

Generation of an EEEV-specific memory immune response was assessed by evaluation for EEEV antigen-specific IgG production from memory B cells at the latest time point available post-transplant and at baseline for comparison. To determine whether cross-reactivity with alphaviruses more commonly associated with human disease could account for any positive results, we also assessed for IgG-producing memory B cells to recombinant envelope protein from chikungunya virus. On DOI 48, 1.34% of total IgG-secreting memory B cells demonstrated reactivity with inactivated EEEV antigen (Figure 2). IgG production from memory B cells was absent in cells stimulated with normal mouse suckling brain antigen or recombinant envelope protein from chikungunya virus (Supplementary Figure 1).

### EEEV-Specific T Cell Responses

**FIGURE 3:**
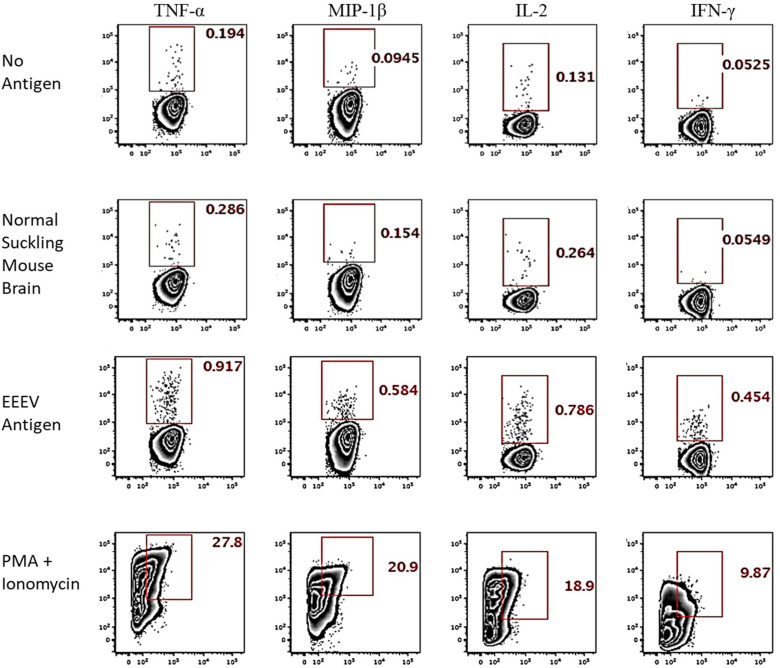
Intracellular Cytokine Staining of CD4+ T cells for TNF-α, MIP-1β, IL-2, and IFN-γ. The two top rows represent negative controls (stimulation with no antigen or normal suckling mouse brain). The third row shows results for stimulation with whole-killed EEEV antigen from EEEV-infected suckling mouse brain demonstrating increased production of all four cytokines from CD4+ T cells when stimulated with EEEV antigen compared to negative controls. The bottom row is the positive control.

**FIGURE 4:**
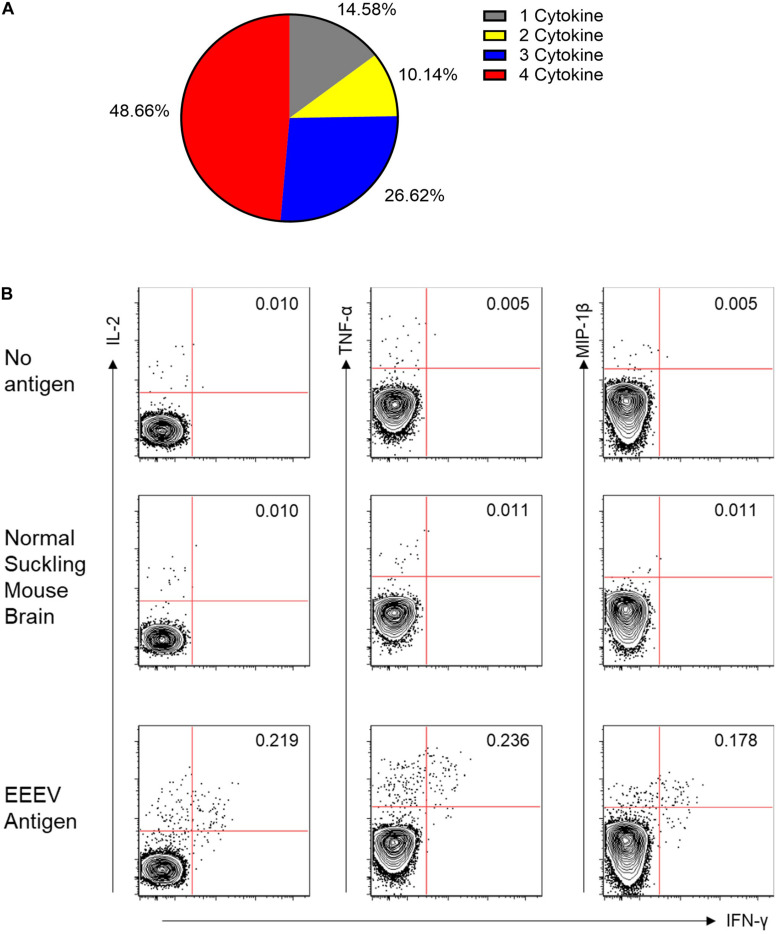
**(A)** Functionality of the CD4+ T Cell Response to EEEV Antigen (TNF-α, MIP-1β, IL-2, and IFN-γ). A high percentage of the observed responding cells (∼85%) represented polyfunctional CD4+ T cells secreting more than one cytokine and were considered to be high quality CD4+ T cell responses. **(B)** Co-Expression of Cytokines from IFN-γ Producing CD4+ T Cells. The top two rows are negative controls using no stimulating antigen and normal mouse suckling brain antigen. The bottom row is the response to EEEV-infected mouse suckling brain preparation (whole-inactivated EEEV). The most commonly co-expressed cytokines were IFN-γ and TNF-α followed by IFN-γ and IL-2.

Assessment of T cell recognition of inactivated EEEV antigen was performed using intracellular cytokine staining for four cytokines: TNF-α, IL-2, MIP-1β, and IFN-γ. Stimulation with EEEV peptides was not performed due to the limited quantity of PBMCs available. Upon stimulation with inactivated EEEV antigen, CD4+ T cells demonstrated increased expression of TNF-α, IL-2, MIP-1β, and IFN-γ (Figure 3). The fold-increase in cytokine expression for EEEV antigen relative to normal mouse suckling brain antigen was highest for IFN-γ (8.27 fold) followed by MIP-1β (3.79 fold), TNF-α (3.21 fold), and IL-2 (2.98 fold). Most CD4+ T cells displayed polyfunctional cytokine production (Figure 4A) with 10% producing two cytokines, 27% producing three cytokines, and 49% producing all four cytokines. Among dual functional CD4+ T cells producing IFN-γ, the most commonly co-produced cytokine was TNF-α followed by IL-2 (Figure 4B). No increase in cytokine expression was observed from CD8+ T cells upon stimulation with inactivated whole EEEV antigen despite appropriate CD8+ T cell stimulation with PMA and ionomycin (Supplementary Figure 2).

### T Cell Epitope Prediction

Epitope prediction utilizing the MHC allele information obtained prior to the patient’s first liver transplant yielded sixty-four distinct predicted 9-mer epitope sequences restricted by at least one of the patient’s MHC class I alleles. Modeling based on the patient’s MHC class II alleles yielded forty-five unique 15-mer predicted epitopes. Predicted epitopes were superimposed on to the one hundred and forty-one 15-mer peptides spanning the EEEV envelope proteins E1 and E2 used for T cell epitope mapping. The predicted MHC class I epitopes spanned fifty-five peptides, including thirty-six sequences in the E1 protein and twenty-eight sequences in the E2 protein, while the MHC class II predicted epitopes spanned thirty peptides, including nineteen sequences in E1 and twenty-six sequences in E2 (Supplementary Table 1).

### T Cell Epitope Mapping

**FIGURE 5:**
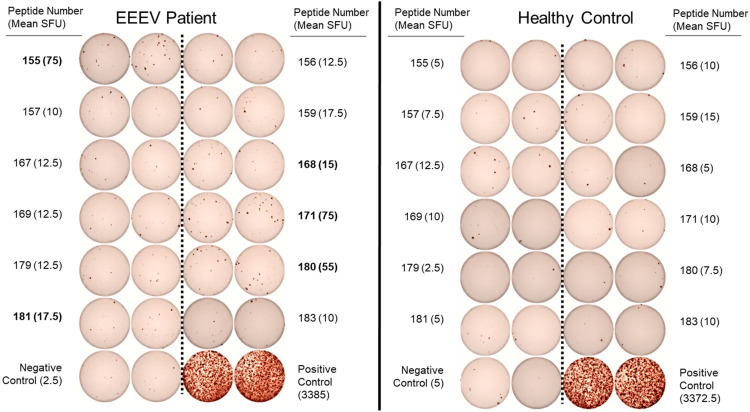
Interferon-γ T Cell ELISpot Used for T Cell Epitope Mapping of Selected Individual Peptides in the EEEV E2 Envelope Protein. Individual peptides were selected following identification in positive peptide pools on the initial screening ELISpot assay. Assays were performed in duplicate for each peptide. Criteria for positivity was defined as mean spot forming unit (SFU) in the EEEV patient at least three times the mean SFU compared to the corresponding peptide response in the healthy control and both negative controls (minimum positive mean SFU: 15 spots). Individual peptides meeting criteria for positivity are listed in bold (peptide numbers 155, 168, 171, 180, and 181).

One hundred and forty-one 15-mer peptides with 10 amino acid overlaps spanning the EEEV envelope proteins E1 and E2 were used for T cell epitope mapping. Forty-eight peptide pools, each consisting of three to six peptides, were generated for an initial screening ELISpot assay. Each peptide was present in two separate, non-overlapping pools. A total of twelve peptide pools were positive on the initial screening ELISpot assay, from which twelve individual peptides (#155, 156, 157, 159, 167 168, 169, 171, 179, 180, 181, and 183) were identified as potential epitopes. Interestingly, each of the twelve potential epitopes was from the E2 envelope protein. These peptides were assessed individually for T cell recognition using T cell ELISpot for the EEEV patient and an otherwise healthy adult control. Six peptides (#155, 159, 168, 171, 180, and 181) elicited positive reactions compared to the negative control but one of these peptides (#159) also elicited a positive response in a healthy control patient compared to the negative control. Peptide #159 was therefore not considered a true positive; all remaining peptides elicited at least three times as many spots in the EEEV patient compared to the healthy control and were considered true positive T cell responses (Figure 5). Three of the positive peptide sequences (#171, 180, and 181) contained both predicted MHC-I and MHC-II epitopes, thus likely stimulating both CD4+ and CD8+ T cells. One sequence (#168) contained only an MHC-I predicted epitope, likely stimulating only CD8+ T cells, and another sequence (#155) contained only an MHC-II predicted epitope, likely stimulating only CD4+ T cells. Due to the limited quantity of PBMCs available, we were unable to separately assess CD4+ and CD8+ T cell responses to individual peptides.

## Discussion

Prior to the cluster of EEEV infections associated with organ transplantation, only two case reports describe EEEV infection among patients receiving immunosuppressive medicine; in both instances, the patients received rituximab prior to developing rapidly fatal EEEV encephalitis without mounting an antibody response (Berlin et al., 2017; Solomon et al., 2017). Evidence from non-human primate and mouse models suggest that antibodies play a role in both protection against disease and clearance of neuroinvasive alphavirus infections, such as EEEV and VEEV in non-human primates and Sindbis virus (SINV) in mice. Passive antibody transfer and monoclonal antibody studies demonstrate protection against infection or development of severe disease when given prior to exposure or up to 48 h post-challenge (Goodchild et al., 2011; Hunt et al., 2011; Phillips et al., 2014; Gardner et al., 2017; Burke et al., 2019; Kim et al., 2019; Ko et al., 2019) and treatment with hyperimmune serum mediates clearance of SINV from neurons *in vitro* and *in vivo* in SINV-infected mice with severe combined immunodeficiency (Levine et al., 1991; Yun et al., 2009; Griffin, 2010). Although the central nervous system is considered an immunoprivileged site, antibody-secreting B cells and virus-specific antibody can be detected in mice with SINV infection (Metcalf and Griffin, 2011; Metcalf et al., 2013).

In contrast to patients on rituximab who were infected with EEEV reported in the literature (Berlin et al., 2017; Solomon et al., 2017), our participant developed antibodies to EEEV despite the use of immunosuppression. The nature of the immunosuppressive agents may contribute to this difference; following pneumococcal vaccine challenge, rituximab has a clear association with an impaired antibody response (van Aalst et al., 2018) whereas solid organ transplant recipients on immunosuppression develop antibody levels comparable to healthy controls (Dendle et al., 2018). The antibody titers observed in this instance of transplant-associated EEEV infection are in line with those from two individual case reports in immunocompetent hosts, although the IgM titers are lower than those reported in two case series (Calisher et al., 1986a, b; Golomb et al., 2001; Garlick et al., 2016). Due to biosafety limitations, specimens from early time points post-infection could not be obtained, although previously published testing reported this individual had detectable serum IgM and neutralizing antibodies present as early as 9 days post-transplantation (Pouch et al., 2018). In previous reports, EEEV IgM was detected as early as 1 day after the onset of symptoms, peaks 1–3 weeks after the onset of illness, and may remain positive for up to 3 months (Calisher et al., 1986a, b). The patient in this study had onset of encephalitis symptoms on DOI 7 (Pouch et al., 2018), making the observed decline in IgM between DOI 39 and DOI 48 consistent with a peak titer occurring in the first 3 weeks after symptom onset. Unfortunately, residual cerebrospinal fluid (CSF) samples were not available for testing from this EEEV patient to document the longitudinal course of CSF antibody production, although clinical testing revealed a positive CSF IgM antibody test on DOI 9 (Pouch et al., 2018).

IgG antibodies to EEEV have been documented as early as 10–11 days after symptom onset in previous cases (Calisher et al., 1986a), unfortunately early specimens were not available from this individual to assess when the IgG response began although rising IgG titers were demonstrated between DOI 39 and DOI 48. No cross-neutralization of WEEV or VEEV were observed despite very high levels of EEEV neutralizing antibodies. This is consistent with data from EEEV, WEEV, and VEEV vaccine development in which, following administration of a viral-like particle representing a single alphavirus, neutralizing antibodies developed only to the corresponding virus with no cross-reactive neutralization observed (Ko et al., 2019). Both the E1 and E2 envelope protein sequences of EEEV differ significantly from the other encephalitic alphaviruses with only 49% and 58% sequence homology in the E1 protein, and 44% and 46% sequence homology in the E2 protein compared to WEEV and VEEV, respectively (Hahn et al., 1988).

For the first time in humans to our knowledge, we describe the cellular immune response following EEEV infection. We observed low absolute numbers of lymphocytes, CD4+ T cells, B cells, monocytes, NK cells, and dendritic cells, which could have been due to EEEV infection, immunosuppressive medication administration, or a combination of both. The absolute CD8+ T cell count was preserved but there was inversion of the CD4:CD8 T cell ratio. Following uncomplicated liver transplantation, the expected changes in immune cell levels and activation status in blood have not been well characterized. One study of uncomplicated liver transplant patients who received steroids and tacrolimus with or without mycophenolate mofetil, demonstrated a mean CD4+ T cell count of 280 cells/μL ± 114 up to 14 days after transplant (Li et al., 2013). Average CD4+ T cell counts were lower among transplant recipients with infection while CD4:CD8 T cell ratios (average ratio 1.02) were similar among uncomplicated and infected liver transplant recipients (Li et al., 2013). In another study of liver transplant recipients, the average absolute counts were low for total lymphocytes (851 cells/μL), CD4+ T cells (388 cells/μL), and CD8+ T cells (306 cells/μL) with an average CD4/CD8 ratio of 1.28 (Kim et al., 2017). These studies suggest while immunosuppression likely contributed to low levels of lymphocytes and CD4+ T cells in this patient, EEEV infection may have raised the absolute number of CD8+ T cells, resulting in an absolute value within the normal range and contributing to inversion of the CD4:CD8 T cell ratio.

Despite depressed absolute levels of many immune cells in the blood, the patient was able to develop an EEEV-specific cellular immune response. On DOI 48, the patient demonstrated a robust IgG memory B cell response to EEEV antigen, which is notable as the absence of increased memory B cell responses has been documented following influenza vaccination among solid organ transplant recipients (Héquet et al., 2016). We concluded this response was secondary to EEEV infection rather than cross-reactivity with other alphaviruses, as the lack of neutralizing antibodies to VEEV and WEEV and lack of IgG-producing memory B cell response to chikungunya envelope protein make it unlikely that the patient was previously infected with other neuroinvasive alphaviruses or one of the most frequently encountered human arthrogenic alphaviruses.

Observation of an EEEV T cell response in this liver transplant recipient on immunosuppression is consistent with literature on T cell responses in solid organ transplant patients, in which influenza vaccination or infection can elicit monofunctional or polyfunctional T cell responses (Héquet et al., 2016; L’Huillier et al., 2020). The EEEV antigen-specific CD4+ T cell response produced by this individual was predominated by polyfunctional cytokine producing cells with greater production of IFN-γ compared to IL-2, MIP-1β, and TNF-α. IFN-γ has been shown to have antiviral activity against EEEV *in vitro* resulting in 10- to 26-fold decreases in viral titers in Vero cells at 24 h and 2- to 8-fold decreases at 72 h (Aguilar et al., 2005). However, two *in vivo* mouse studies suggest IFN-γ may not play a significant role in control of the infection; one study demonstrated similar levels of viremia and mortality in EEEV-infected IFN-γ deficient mice compared to wild-type mice while another demonstrated IFN-γ played only a minor role in extending of the average survival time (Aguilar et al., 2005; Gardner et al., 2009). These studies suggest that despite significant increases in IFN-γ production seen in this patient, IFN-γ may not have played a predominant role mediating this individual’s recovery from EEEV. CD4+ T cells play an important immunological role in animal models of neuroinvasive alphavirus disease, with adoptive transfer of CD4+ T cells providing protection against lethal VEEV encephalitis in αβ T cell receptor knock-out mice and being the primary producers of IFN-γ in the central nervous system in mouse models of SINV and VEEV (Rowell and Griffin, 2002; Yun et al., 2009; Brooke et al., 2010). However, the importance of CD4+ and CD8+ T cells in central nervous system disease in animal models of neuroinvasive infection is unclear, with one study demonstrating these cells assist in clearance from the central nervous system of mice while another demonstrated protection from lethal encephalitis following knock-out of CD4+ T cells and CD8+ T cells (Rowell and Griffin, 2002; Brooke et al., 2010).

T cell priming with whole protein antigens primarily activates CD4+ T cells over CD8+ T cells whereas peptide antigen stimulation can activate both cell types (Zhang et al., 2009). As expected using a whole protein antigen, we observed no EEEV-specific CD8+ T cell response by intracellular cytokine staining following inactivated EEEV antigen stimulation of PBMCs. Unfortunately due to limited PBMC quantities, we were unable to perform intracellular cytokine staining using EEEV envelope peptides for stimulation to profile the nature of the EEEV-specific CD8+ T cell response.

Although T cell epitope prediction yielded predicted epitopes in both the EEEV E1 and E2 proteins, epitope mapping only identified T cell responses to peptides in the E2 protein. All individual peptide eliciting a positive T cell response by ELISpot contained epitopes that were predicted based on the patient’s MHC class I and/or class II alleles. It is possible that additional T cell epitopes were missed due to the experimental methodology used in this study. We chose to use the IFN-γ ELISpot technique for epitope mapping due to limited PBMC quantities and evidence that IFN-γ was the predominant cytokine produced by CD4+ T cells in response to EEEV antigen, although we were not able to assess how IFN-γ is representative of CD8+ T cell EEEV recognition. If EEEV infection elicits weak IFN-γ response from CD8+ T cells, our epitope mapping results may preferentially recognize CD4+ T cell epitopes and under recognize CD8+ T cell epitopes. Additional limitations of using this technique include limitation of responses to linear peptide sequences rather than conformational epitopes and epitopes recognized by T cells producing cytokines other than IFN-γ were not identified. Identifying immunogenic epitopes on EEEV may assist with informing EEEV vaccine design. Two EEEV vaccine candidates, a whole-inactivated EEEV vaccine and a trivalent EEEV/WEEV/VEEV virus-like particle vaccine, have already advanced into human clinical trials.

We recognize that the data presented reflects results from a single EEEV-infected individual who was on immunosuppression at the time infection and acquired EEEV by a non-traditional route, therefore may not be representative of the immune responses in immunocompetent individuals who acquire EEEV via the bite of an infected mosquito. Additionally, our ability to fully characterize the immune response to this infection was limited due to PBMCs quantities and by the limited time points available for collection. Despite these limitations, this work serves as a starting point for improving our understanding of the immune response to EEEV, as to our knowledge this is the first available data on the *in vivo* human cellular immune response to EEEV or other encephalitis alphaviruses. Additional studies of the cellular immune response to the EEEV in humans are needed to determine if the findings reported in this paper are generalizable, for providing data for the rational design of future EEEV vaccine candidates, to delineate differences in the immune response among fatal versus non-fatal human EEEV infections, and to shed light on why infection results in severe disease in some people, but asymptomatic or mild disease in others. Given the rarity of EEEV infection, this will require pre-emptive planning and the establishment of close collaborations between clinicians and translational researchers to rapidly identify and mobilize existing resources to engage patients in research opportunities. The significantly increased number of patients with EEEV disease observed in the United States in 2019 demonstrates both the importance and an opportunity for continuing to broaden our knowledge of human immune responses to EEEV (Morens et al., 2019).

## Data Availability Statement

The raw data supporting the conclusions of this article will be made available by the authors, without undue reservation.

## Ethics Statement

The studies involving human participants were reviewed and approved by the Emory University Institutional Review Board. The legally authorized representative of the patients/participants provided their written informed consent to participate in this study.

## Author Contributions

VR contributed to designing the research studies, conducting the experiments, acquiring and analyzing the data, and writing the manuscript. LL, CB, AP, and CG contributed to conducting the experiments, acquiring and analyzing the data, and writing the manuscript. YX, CH, and DW contributed to conducting the experiments and acquiring and analyzing the data. SP contributed to acquiring the data and writing the manuscript. PG and MM contributed to designing the research studies, analyzing the data, and writing the manuscript. All authors contributed to the article and approved the submitted version.

## Conflict of Interest

The authors declare that the research was conducted in the absence of any commercial or financial relationships that could be construed as a potential conflict of interest.
