# Supplementary material for: The Immune Response to Eastern Equine Encephalitis Virus Acquired Through Organ Transplantation
Source: Front Microbiol. 2020 Sep 24;11:561530. doi: 10.3389/fmicb.2020.561530 (PMC7541818; doi:10.3389/fmicb.2020.561530)
Supplement: Supplementary file 1 [file Data_Sheet_1.docx]

Supplementary Material

# Supplementary Figures and Tables

**Supplementary Figure 1.** **Supplementary Figure 1:** Memory B Cell ELISpot Response to Mouse Suckling Brain and Recombinant Chikungunya Glycoprotein on DOI 48


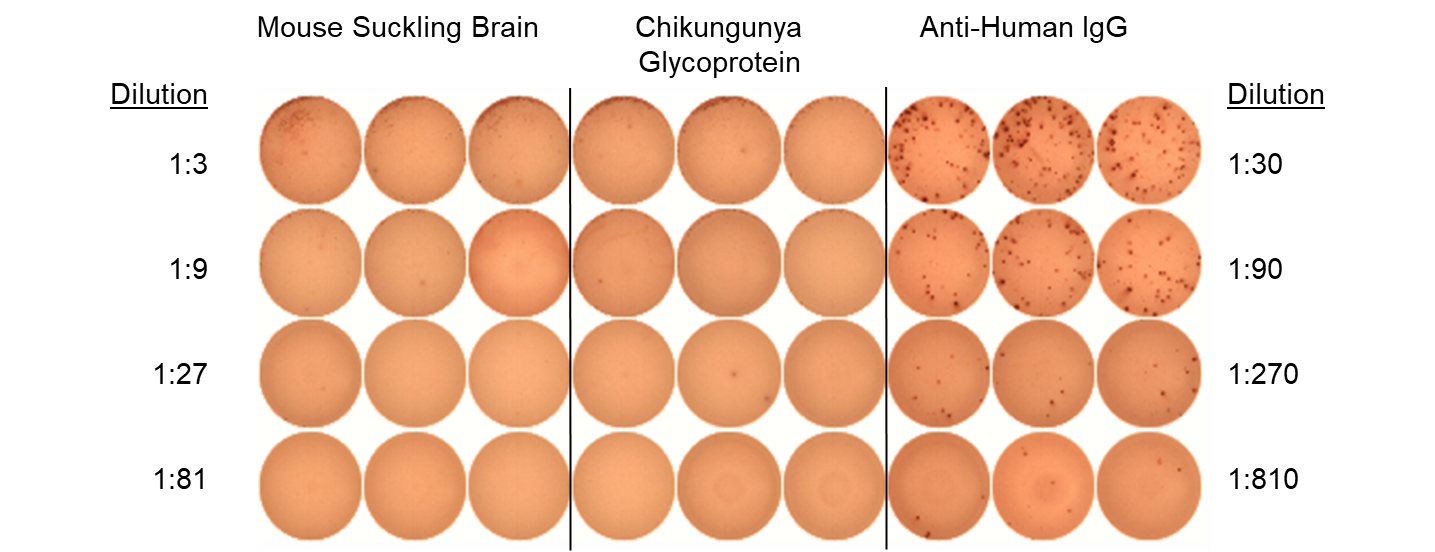


Dilution

1:30

1:90

1:270

1:810

IgG-producing memory B cells that recognized mouse brain antigen or recombinant Chikungunya envelope protein were absent in the EEEV patient.

**Supplementary Figure 2:** Intracellular Cytokine Staining for TNF-α, MIP-1β, IL-2, and IFN-γ from CD8+ T Cells


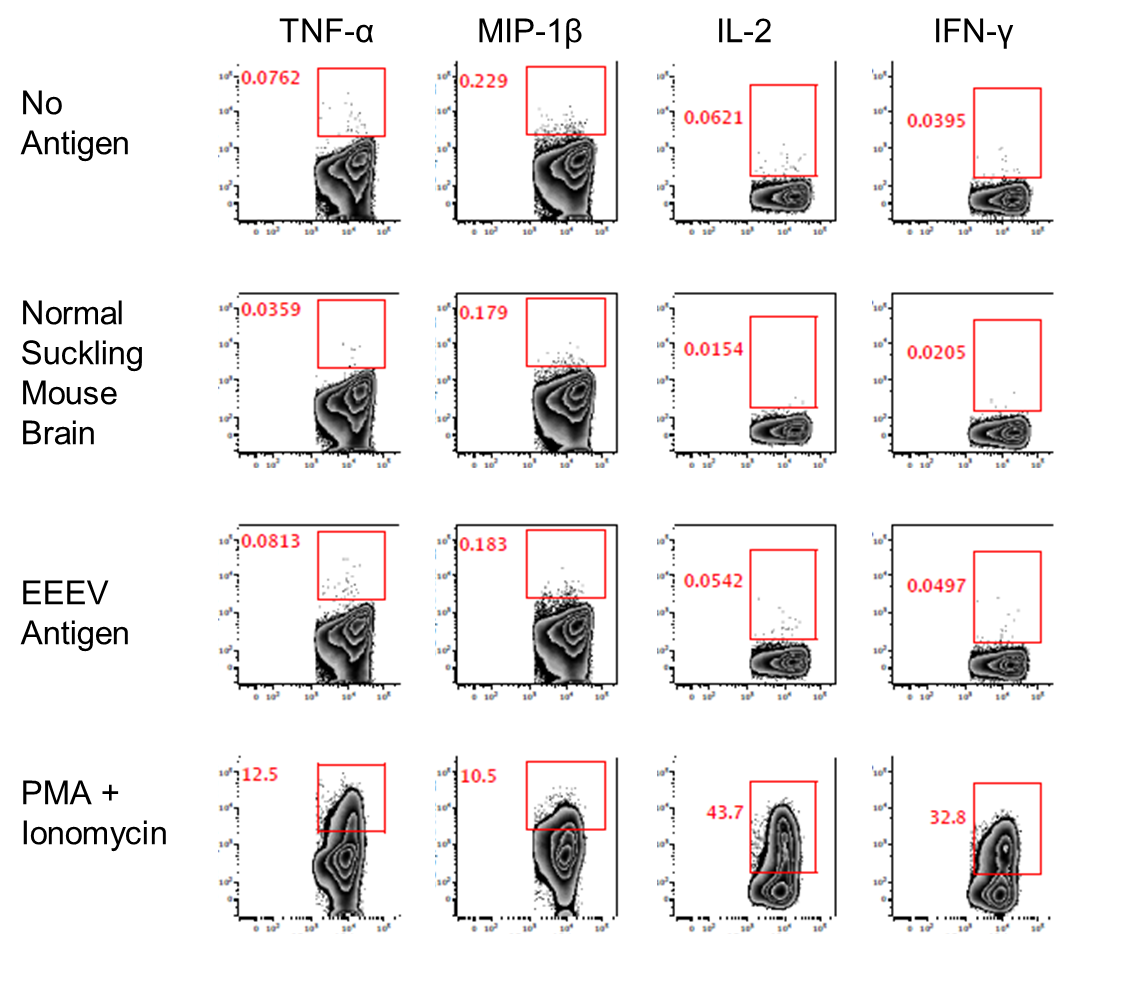


The two top rows represent negative controls (stimulation with no antigen or normal suckling mouse brain). The third row shows results for stimulation with whole-killed EEEV antigen from EEEV-infected suckling mouse brain demonstrating no increase in production of any of the four cytokines from CD8+ T cells compared to negative controls. The bottom row is the positive control.

## Supplementary Table 1: Eastern Equine Encephalitis Virus Envelope Protein 1 and 2 Peptide Sequences and Predicted MHC-I Restricted Epitope Sequences

| **Envelope Domain** | **Peptide Number** | **Sequence** | **MHC-I Predicted Epitope Sequence(s)** |
| --- | --- | --- | --- |
| 2 | 143 | DLDTHFTQYKLARPY | HFTQYKLAR |
| 2 | 144 | TQYKLARPYIADCPN | - |
| 2 | 145 | RPYIADCPNCGHSRC | - |
| 2 | 146 | CPNCGHSRCDSPIAI | - |
| 2 | 147 | SRCDSPIAIEEVRGD | - |
| 2 | 148 | IAIEEVRGDAHAGVI | - |
| 2 | 149 | RGDAHAGVIRIQTSA | - |
| 2 | 150 | GVIRIQTSAMFGLKT | - |
| 2 | 151 | TSAMFGLKTDGVDLA | - |
| 2 | 152 | LKTDGVDLAYMSFMN | - |
| 2 | 153 | DLAYMSFMNGKTQKS | - |
| 2 | 154 | FMNGKTQKSIKIDNL | - |
| **2** | **155** | **QKSIKIDNLHVRTSA** | - |
| 2 | 156 | DNLHVRTSAPCSLVS | VRTSAPCSL |
| 2 | 157 | TSAPCSLVSHHGYYI | LVSHHGYYI |
| 2 | 158 | LVSHHGYYILAQCPP | LVSHHGYYI |
|  |  |  | VSHHGYYIL |
| 2 | 159 | YYILAQCPPGDTVTV | - |
| 2 | 160 | CPPGDTVTVGFHDGP | - |
| 2 | 161 | VTVGFHDGPNRHTCT | - |
| 2 | 162 | DGPNRHTCTVAHKVE | - |
| 2 | 163 | TCTVAHKVEFRPVGR | - |
| 2 | 164 | KVEFRPVGREKYRHP | FRPVGREKY |
| 2 | 165 | VGREKYRHPPEHGVE | YRHPPEHGV |
| 2 | 166 | RHPPEHGVELPCNRY | - |
| 2 | 167 | GVELPCNRYTHKRAD | NRYTHKRAD |
| **2** | **168** | **NRYTHKRADQGHYVE** | **NRYTHKRAD** |
|  |  |  | **KRADQGHYV** |
| 2 | 169 | RADQGHYVEMHQPGL | YVEMHQPGL |
| 2 | 170 | YVEMHQPGLVADHSL | YVEMHQPGL |
| **2** | **171** | **PGLVADHSLLSIHSA** | **VADHSLLSI** |
| 2 | 172 | HSLLSIHSAKVKITV | HSAKVKITV |
|  |  |  |  |
| 2 | 173 | HSAKVKITVPSGAQV | HSAKVKITV |
|  |  |  | ITVPSGAQV |
| 2 | 174 | ITVPSGAQVKYYCKC | ITVPSGAQV |
| 2 | 175 | AQVKYYCKCPDVREG | YYCKCPDVR |
| 2 | 176 | CKCPDVREGITSSDH | - |
| 2 | 177 | REGITSSDHTTTCTD | - |
| 2 | 178 | SDHTTTCTDVKQCRA | - |
| 2 | 179 | CTDVKQCRAYLIDNK | - |
| **2** | **180** | **CRAYLIDNKKWVYNS** | **YLIDNKKWV** |
| **2** | **181** | **DNKKWVYNSGRLPRG** | **VYNSGRLPR** |
| 2 | 182 | YNSGRLPRGEGDTFK | - |
| 2 | 183 | PRGEGDTFKGKLHVP | - |
| 2 | 184 | TFKGKLHVPFVPVKA | - |
| 2 | 185 | HVPFVPVKAKCIATL | - |
| 2 | 186 | VKAKCIATLAPEPLV | IATLAPEPL |
| 2 | 187 | ATLAPEPLVEHKHRT | - |
| 2 | 188 | PLVEHKHRTLILHLH | - |
| 2 | 189 | HRTLILHLHPDHPTL | HLHPDHPTL |
| 2 | 190 | HLHPDHPTLLTTRSL | HLHPDHPTL |
| 2 | 191 | PTLLTTRSLGSDANP | - |
| 2 | 192 | RSLGSDANPTRQWIE | - |
| 2 | 193 | ANPTRQWIERPTTVN | - |
| 2 | 194 | WIERPTTVNFTVTGE | - |
| 2 | 195 | TVNFTVTGEGLEYTW | - |
| 2 | 196 | TGEGLEYTWGNHPPK | - |
| 2 | 197 | YTWGNHPPKRVWAQE | - |
| 2 | 198 | PPKRVWAQESGEGNP | - |
| 2 | 199 | AQESGEGNPHGWPHE | - |
| 2 | 200 | GNPHGWPHEVVVYYY | HGWPHEVVV |
| 2 | 201 | PHEVVVYYYNRYPLT | EVVVYYYNR |
|  |  |  | YYYNRYPLT |
| 2 | 202 | YYYNRYPLTTIIGLC | YYYNRYPLT |
|  |  |  | NRYPLTTII |
|  |  |  | YPLTTIIGL |
| 2 | 203 | PLTTIIGLCTCVAII | - |
| 2 | 204 | GLCTCVAIIMVSCVT | - |
| 2 | 205 | AIIMVSCVTSVWLLC | IMVSCVTSV |
|  |  |  | MVSCVTSVW |
| 2 | 206 | CVTSVWLLCRTRNLC | SVWLLCRTR |
| 2 | 207 | LLCRTRNLCITPYKL | - |
| 2 | 208 | NLCITPYKLAPNAQV | - |
| 2 | 209 | YKLAPNAQVPILLAL | LAPNAQVPI |
|  |  |  | AQVPILLAL |
| 2 | 210 | AQVPILLALLCCIKP | AQVPILLAL |
|  |  |  | ILLALLCCI |
| 2 | 211 | PILLALLCCIKPTRA | ILLALLCCI |
| 1 | 212 | YEHTAVMPNKVGIPY | AVMPNKVGI |
| 1 | 213 | MPNKVGIPYKALVER | - |
| 1 | 214 | IPYKALVERPGYAPV | - |
| 1 | 215 | VERPGYAPVHLQIQL | - |
| 1 | 216 | APVHLQIQLVNTRII | - |
| 1 | 217 | IQLVNTRIIPSTNLE | - |
| 1 | 218 | RIIPSTNLEYITCKY | - |
| 1 | 219 | NLEYITCKYKTKVPS | - |
| 1 | 220 | CKYKTKVPSPVVKCC | - |
| 1 | 221 | VPSPVVKCCGATQCT | - |
| 1 | 222 | KCCGATQCTSKPHPD | - |
| 1 | 223 | QCTSKPHPDYQCQVF | - |
| 1 | 224 | HPDYQCQVFTGVYPF | QVFTGVYPF |
| 1 | 225 | QVFTGVYPFMWGGAY | QVFTGVYPF |
|  |  |  | FTGVYPFMW |
| 1 | 226 | YPFMWGGAYCFCDTE | FMWGGAYCF |
| 1 | 227 | GAYCFCDTENTQMSE | - |
| 1 | 228 | DTENTQMSEAYVERS | - |
| 1 | 229 | MSEAYVERSEECSID | - |
| 1 | 230 | ERSEECSIDHAKAYK | - |
| 1 | 231 | SIDHAKAYKVHTGTV | - |
| 1 | 232 | AYKVHTGTVQAMVNI | - |
| 1 | 233 | GTVQAMVNITYGSVS | MVNITYGSV |
| 1 | 234 | VNITYGSVSWRSADV | ITYGSVSWR |
| 1 | 235 | SVSWRSADVYVNGET | - |
| 1 | 236 | ADVYVNGETPAKIGD | - |
| 1 | 237 | GETPAKIGDAKLIIG | - |
| 1 | 238 | IGDAKLIIGPLSSAW | - |
| 1 | 239 | IIGPLSSAWSPFDNK | - |
| 1 | 240 | SAWSPFDNKVVVYGH | - |
| 1 | 241 | DNKVVVYGHEVYNYD | - |
| 1 | 242 | YGHEVYNYDFPEYGT | - |
| 1 | 243 | NYDFPEYGTGKAGSF | - |
| 1 | 244 | YGTGKAGSFGDLQSR | - |
| 1 | 245 | GSFGDLQSRTSTSND | - |
| 1 | 246 | QSRTSTSNDLYANTN | SRTSTSNDL |
|  |  |  | RTSTSNDLY |
| 1 | 247 | SNDLYANTNLKLQRP | - |
| 1 | 248 | NTNLKLQRPQAGIVH | - |
| 1 | 249 | QRPQAGIVHTPFTQA | - |
| 1 | 250 | IVHTPFTQAPSGFER | - |
| 1 | 251 | TQAPSGFERWKRDKG | - |
| 1 | 252 | FERWKRDKGAPLNDV | - |
| 1 | 253 | DKGAPLNDVAPFGCS | - |
| 1 | 254 | NDVAPFGCSIALEPL | APFGCSIAL |
| 1 | 255 | GCSIALEPLRAENCA | - |
| 1 | 256 | EPLRAENCAVGSIPI | - |
| 1 | 257 | NCAVGSIPISIDIPD | GSIPISIDI |
| 1 | 258 | IPISIDIPDAAFTRI | ISIDIPDAA |
|  |  |  | DIPDAAFTR |
| 1 | 259 | IPDAAFTRISETPTV | TRISETPTV |
| 1 | 260 | TRISETPTVSDLECK | TRISETPTV |
| 1 | 261 | PTVSDLECKITECTY | - |
| 1 | 262 | ECKITECTYASDFGG | - |
| 1 | 263 | CTYASDFGGIATVAY | YASDFGGIA |
| 1 | 264 | FGGIATVAYKSSKAG | - |
| 1 | 265 | VAYKSSKAGNCPIHS | - |
| 1 | 266 | KAGNCPIHSPSGVAV | - |
| 1 | 267 | IHSPSGVAVIKENDV | HSPSGVAVI |
| 1 | 268 | VAVIKENDVTLAESG | VIKENDVTL |
| 1 | 269 | NDVTLAESGSFTFHF | VTLAESGSF |
|  |  |  | LAESGSFTF |
| 1 | 270 | ESGSFTFHFSTANIH | - |
| 1 | 271 | FHFSTANIHPAFKLQ | FSTANIHPA |
|  |  |  | STANIHPAF |
| 1 | 272 | NIHPAFKLQVCTSAV | KLQVCTSAV |
| 1 | 273 | KLQVCTSAVTCKGDC | KLQVCTSAV |
| 1 | 274 | SAVTCKGDCKPPKDH | - |
| 1 | 275 | GDCKPPKDHIVDYPA | - |
| 1 | 276 | KDHIVDYPAQHTESF | YPAQHTESF |
| 1 | 277 | YPAQHTESFTSAISA | YPAQHTESF |
|  |  |  | HTESFTSAI |
| 1 | 278 | ESFTSAISATAWSWL | FTSAISATA |
|  |  |  | TSAISATAW |
|  |  |  | AISATAWSW |
|  |  |  | ISATAWSWL |
| 1 | 279 | ISATAWSWLKVLVGG | ISATAWSWL |
|  |  |  | TAWSWLKVL |
| 1 | 280 | SWLKVLVGGTSAFIV | VLVGGTSAF |
| 1 | 281 | VGGTSAFIVLGLIAT | TSAFIVLGL |
| 1 | 282 | FIVLGLIATAVVALV | LIATAVVAL |
|  |  |  | IATAVVALV |
|  |  |  | ATAVVALVL |
| 1 | 283 | IATAVVALVLFFHRH | IATAVVALV |
|  |  |  | ATAVVALVL |
|  |  |  | TAVVALVLF |
|  |  |  | AVVALVLFF |
|  |  |  | VALVLFFHR |

*MHC-II predicted epitopes were considered a match to a peptide if at least 10 amino acids of the predicted epitope overlapped with the peptide sequence

Peptides, with the corresponding sequences and predicted epitopes, in bold are those that yielded positive responses on T cell epitope mapping from the EEEV patient.
